# Supplementary material for: Saccharomyces boulardii (CNCM I-745) improves intestinal damage in sepsis by remodeling intestinal flora structure
Source: Front Cell Infect Microbiol. 2025 Jul 31;15:1602792. doi: 10.3389/fcimb.2025.1602792 (PMC12350329; doi:10.3389/fcimb.2025.1602792)
Supplement: Supplementary file 1 [file DataSheet1.zip › summary/summary/8_Advanced_analyse/3_BUGbase_result/taxa_contributions/Anaerobic.pdf]

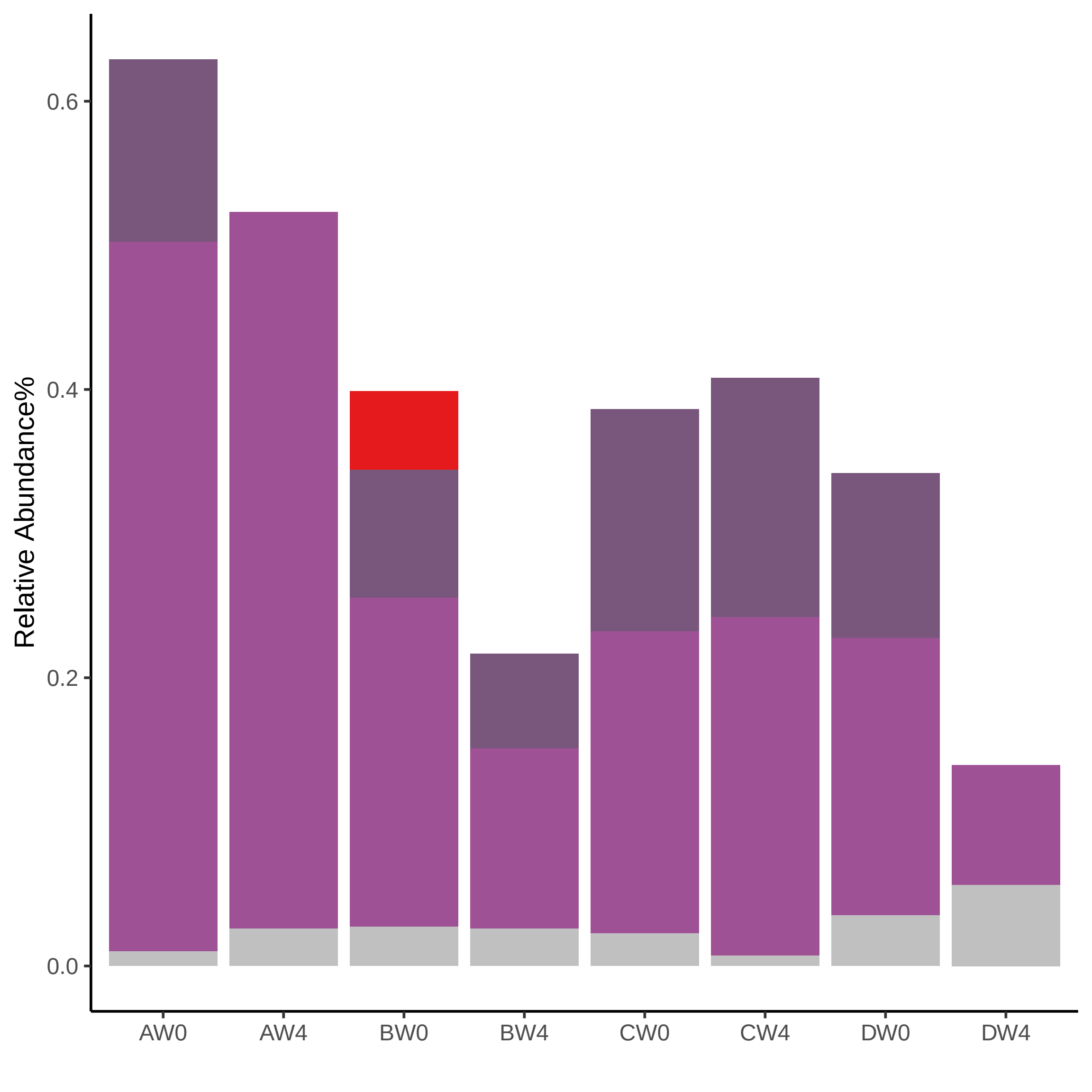
Relative Abundance%
0.6
0.4
0.2
0.0
AW0
AW4
BW0
BW4
CW0
CW4
DW0
DW4
